# HCG (1500IU) administration on day 3 after oocytes retrieval, following GnRH-agonist trigger for final follicular maturation, results in high sufficient mid luteal progesterone levels - a proof of concept

**DOI:** 10.1186/1757-2215-7-35

**Published:** 2014-04-03

**Authors:** Jigal Haas, Alon Kedem, Ronit Machtinger, Shir Dar, Ariel Hourvitz, Gil Yerushalmi, Raoul Orvieto

**Affiliations:** 1 Department of Obstetrics and Gynecology, Chaim Sheba Medical Center (Tel Hashomer), Ramat Gan, Israel; 2 Sackler Faculty of Medicine, Tel Aviv University, Tel Aviv, Israel

**Keywords:** Ovarian hyperstimulation syndrome, Ovarian stimulation, GnRH agonist trigger, hCG rescue, Prevention

## Abstract

**Background:**

Controlled ovarian hyperstimulation (COH) which combining GnRH antagonist co-treatment and GnRH agonist trigger with an additional 1500 IU hCG luteal rescue on day of oocytes retrieval, has become a common tool aiming to reduce severe ovarian hyperstimulation syndrome (OHSS). In the present, proof of concept study, we evaluate whether by deferring the hCG rescue bolus for 3 days, we are still able to rescue the luteal phase.

**Methods:**

Patients undergoing the GnRH-antagonist protocol, who were considered at high risk for developing severe OHSS and received GnRH-agonist for final oocyte maturation, were included. For luteal phase support, all patients received an “intense” luteal support. Those who had no signs of *early* moderate OHSS on day 3 after oocytes retrieval were instructed to inject 1500 IU of HCG bolus (hCG group). Ovarian stimulation characteristics and mid luteal progesterone levels were compared between those who received the HCG bolus and those who did not.

**Results:**

Eleven IVF cycles were evaluated, 5 in the hCG group and 6 in the intense luteal support only group. While no in-between group differences were observed in ovarian stimulation characteristics, significantly higher mid luteal progesterone levels (>127 nmol/L vs 42.1 ± 14.5 nmol/L, respectively) and a non-significant increase in pregnancy rate (40% vs 16.6%, respectively), were observed in those who receive the hCG bolus compared to those who did not.

**Conclusions:**

hCG luteal rescue should be offered 3 days after oocytes retrieval only to those patients with no signs of early moderate OHSS. Further studies are required to elucidate the appropriate regimen of luteal HCG administration in patients undergoing final follicular maturation with GnRH-agonist.

## Background

Ovarian hyperstimulation syndrome (OHSS) is a serious complication of ovulation induction, almost always presents either after human chorionic gonadotropin (hCG) administration in susceptible patients or during early pregnancy. Despite many years of clinical experience, the pathophysiology of OHSS is poorly understood and there is no reliable test to predict patients who will subsequently develop severe OHSS [1]. Of notice, that while investigating randomized controlled trials that aimed to prevent of severe OHSS, we found that different studies defined high-risk patients by different estradiol (E2) threshold levels, ranging from 1906 to 6000 pg/ml, most used a level of ~3000 pg/ml [2]. Moreover, when all the predictive variables for severe OHSS were combined, the prevalence of severe OHSS in the ostensibly high risk patients was only about *20%*[2] an extremely low value for a good predictive measure (false positive-80%).

Furthermore, there are no precise methods to completely prevent severe OHSS, except for withholding the ovulation-inducing trigger of hCG, or replacing hCG with GnRH agonist (GnRHa) to trigger ovulation [3,4]. However, due to the reported significantly reduced clinical pregnancy and increased first trimester pregnancy loss [5,6] using the GnRH antagonist controlled ovarian hyperstimulation (COH) protocol with GnRHa trigger, efforts have been made to improve reproductive outcome, by manipulating the luteal phase. The three suggested optional strategies aiming to improve outcome are freeze-all policy; fresh transfer and intensive luteal support; and fresh transfer and low-dose HCG supplementation [7].

One bolus of 1500 IU hCG 35 h after the triggering bolus of GnRHa (on day of oocytes retrieval) was demonstrated to rescue the luteal phase, resulting in a reproductive outcome comparable with that of HCG triggering, and with no increased risk of OHSS [8,9]. However, in a recently published retrospective study, Seyhan et al. [10] have challenged the safety of the GnRHa trigger with HCG rescue. They examined whether GnRHa trigger and 1500 IU hCG luteal rescue protocol, completely prevented severe OHSS. Of the 23 patients evaluated, 6 (26%) developed severe OHSS, 5 of whom had severe early OHSS requiring ascites drainage and hospitalization. Figure that is comparable to the aforementioned 20% prevalence of severe OHSS, in high risk patients.

Since early OHSS occurs 3–7 days following hCG trigger for final follicular maturation, it is our policy to withhold the 1500 IU hCG luteal rescue bolus and to follow and re-evaluate these patients for 3 days after oocyte retrieval [day of embryo transfer (ET)] for signs of *early* moderate OHSS. Only those patients with no signs of early OHSS are instructed to inject 1500 IU of HCG. By deferring the hCG bolus by 3 days (5 days following GnRHa trigger), we believe that we are actually offering the hCG to 80% of the *a priori* at risk patients (the aforementioned- detection rate c’ 80% false positive), who are not supposed to develop severe early OHSS, while avoiding hCG luteal rescue to the “real” 20-26% [2,10] patient at risk to develop severe early-OHSS.

The main criticism of the aforementioned protocol, that we aimed to challenge in the present proof of concept study, is whether by deferring the hCG rescue bolus for 3 days, we are still able to rescue the luteal phase, as evident by sufficient mid luteal progesterone levels, without jeopardizing pregnancy rate or increasing the risk for severe OHSS.

## Methods

We reviewed the computerized files of all consecutive women admitted to our in-vitro fertilization (IVF) unit from August 2013 to December 2013 who reached the oocyte retrieval stage. Only those patients undergoing the multiple-dose GnRH-antagonist protocol, who were considered at high risk of developing severe OHSS, i.e. those with rapidly rising serum E2 levels; peak E2 level in excess of 9175 pmol/L (2,500 pg/mL); and/or the emergence of a large number of intermediate sized follicles [11] and received 0.2 mg triptorelin (Decapeptyl, Ferring, Malmo, Sweden) for final oocyte maturation, were included. The study was approved by our institutional review board (IRB number 0776-13-SMC).

For luteal phase support, all patients received an “intense” luteal support [12], starting 1 day after oocyte retrieval, that included 4 mg daily E2 valerate per os (Progynova; Schering), combined with either 50 mg progesterone IM (Gestone, Ferring- Lapidot, Israel) daily, 400 mg micronized progesterone vaginal tablets (Endometrin, Ferring-Lapidot, Israel) in two divided doses, or 180 mg micronized progesterone vaginal gel (Crinone® 8%, Merck Serono, Herzelia, Israel) in two divided doses.

Three days after oocyte retrieval (day of ET), patients were evaluated for signs of *early* moderate OHSS (ultrasonographic signs of ascites as reflected by the appearance of fluid surrounding the uterus/ovaries, and/or hematocrit levels >40% for the degree of haemoconcentration). If signs of early moderate OHSS appeared, one embryo was transferred, and the patient was instructed to continue with the intense luteal support only (Intense support only group). If no early signs of OHSS developed, one embryo was transferred, and the patient was instructed to continue with the intense luteal support and to inject 1500 IU of HCG (hCG group).

According to Engmann’s protocol [12], serum progesterone levels were measured 4 days after embryo transfer (One week after oocyte retrieval- mid luteal phase), and additional IM progesterone was provided to increase serum progesterone levels if needed.

Data on patient demographics, controlled ovarian hyperstimulation (COH) characteristics and mid luteal progesterone levels were recorded and compared between those who received a bolus of 1500 IU hCG on day of ET (hCG group) and those who did not (Intense support only group).

Results are presented as means ± SD. Differences in variables between the two luteal different luteal supports were statistically analyzed with Student’s t-test and Chi-squared test, as appropriate. A p value of less than 0.05 was considered significant.

## Results

Eleven IVF cycles in 11 women were evaluated, 5 in the hCG group (age 31.6 ± 4.6) and 6 in the intense luteal support only group (age 29.8 ± 5.1). The clinical characteristics of the IVF cycles in the two study groups are shown in Table 1.

**Table 1:** Comparison between IVF cycles with intense luteal support only versus additional luteal hCG

	**hCG group (n = 5)**	**Intense luteal support group (n = 6)**	**p values**
Age (yrs)	31.6 ± 4.6	29.8 ± 5.1	ns
BMI (kg/m2)	23.65 ± 3.8	23.0 ± 2.5	ns
Length of stimulation (days)	9 ± 2.1.8	10.3 ± 3.8	ns
Number of gonadotropin ampoules used	17.1 ± 3.4	22.0 ± 11.5	ns
Peak E2 levels on day of hCG administration (pmol/L)	10597 ± 669	13064 ± 3019	ns
Peak progesterone levels on day of hCG administration (nmol/L)	2.1 ± 0.2	2.3 ± 0.5	ns
Number of oocytes retrieved	18.4 ± 3.5	19.6 ± 3.0	ns
Mid-luteal progesterone levels (nmol/L)	>127	42.1 ± 14.5	<0.0001
Pregnancy rate	2/5(40%)	1/6(16.6%)	ns

While no in-between group differences were observed in patients’ body mass index, the length of ovarian stimulation, number of gonadotropin ampoules used, peak E2 and progesterone levels on day of hCG administration, nor in the number of oocytes retrieved (Table 1), mid-luteal progesterone levels were significantly higher in those who receive a 1500 IU hCG bolus, as compared to those who did not (>127 nmol/L vs 42.1 ± 14.5 nmol/L; p < 0.0001, respectively).

Pregnancy was achieved in 2 patients in the hCG group (40%) and one in the intense support only group (16.6%); this difference wasn’t statistically significant. Regarding severe OHSS, no case of severe OHSS was reported among the study groups.

## Discussion

The key to preventing OHSS is experience with ovulation stimulation therapy and recognition of “risk factors” for OHSS. In our practice, normal and high-responder patients undergoing their first IVF attempt are offered the COH with a GnRH antagonist [13]. With this strategy we are able to substitute hCG with the GnRHa to trigger ovulation in those at risk to develop severe OHSS [11].

In those achieving ≥20 oocytes, the freeze all policy with the subsequent frozen-thawed ET, is usually recommended. However, if less than 20 oocytes are retrieved, patients are instructed to start an intensive luteal support with estradiol and progesterone and are evaluated 3 days after oocyte retrieval for signs of *early* moderate OHSS. A bolus of 1500 IU hCG is offered *only* to those with no signs of early moderate OHSS.

In this small series of 11 patients, we demonstrated that by deferring the hCG bolus by 3 days (5 days following GnRHa trigger, instead of day of oocytes retrieval), we are still able to rescue the corpus luteum, with an observed extremely high midluteal progesterone levels and with no cases of severe OHSS. This observation is in accordance with previous studies, in the human as well as the animal model, that demonstrated corpus luteum rescue after three days of LH deprivation [14,15]. Moreover, in their elegant rhesus monkeys model, Hutchinson and Zeleznik have demonstrated the restoration of gonadotropin secretion following 3-day gonadotropin deprivation period, during the early (days 2–5) and mid (days 8–11) luteal phase (but not in the late ≥ 13 days), that resulted in resumption of progesterone secretion which continued for a period of time which effectively completed the typical lifespan of the corpus luteum [15].

The mid luteal progesterone levels obtained in our study, in the intense support only group were 42.1 ± 14.5 nmol/L. Figure, that is in the lower range for successful implantation [16]. Moreover, the mid luteal progesterone levels obtained in our study, in patients receiving the hCG luteal bolus 3 days following oocytes retrieval, are comparable to the levels obtained in those receiving the hCG luteal bolus on the day of oocytes retrieval [17].

Moreover, we believe that by doing so, we are actually offering the hCG to 80% of the *a priori* at risk patients (the aforementioned- detection rate c’ 80% false positive), who are not supposed to develop severe early OHSS, while avoiding hCG administration to the “real” 20-26% [2,10] patient at risk to develop severe early-OHSS. Despite a rather limited experience, no case of OHSS has been encountered and a reasonable pregnancy rate was achieved (40%).

Following the study by Seyhan et al. [10], demonstrating an extremely high prevalence of severe OHSS following 1500 IU hCG bolus on day of oocytes retrieval, it seems that those patients triggered by GnRHa for final oocytes maturation, remain with two treatment options: (a) the freeze-all policy- notwithstanding the recent improvement in live birth rates after replacement frozen-thawed vitrified oocytes/embryos, it should be emphasized, that in most centers, there is still a gap in live birth rates between fresh and frozen/thawed cycles (in favor of fresh cycle); and (b) intensive luteal support of estradiol and progesterone- the data regarding the efficacy of this modality are still inconclusive. While comparing our experience with GnRHa trigger before [6] and after modifying our luteal-phase support to the intensive support with E2 and progesterone, we could not demonstrate any differences in peak E2 levels, fertilization rate, number of embryos transferred or implantation and pregnancy rates, between the two luteal support regimens [18]. Moreover, in both groups of luteal support following GnRH-a trigger, implantation and pregnancy rates were lower compared to HCG trigger [6,18]. Therefore, the search for an optimal luteal support regimen to patients receiving GnRHa trigger should be continued.

In the present study, with its inherent limitation due to the small sample size, we offer a new treatment modality that clearly supports the concept of rescuing the corpus luteum, despite the 3 days delay in hCG administration (3 days following oocytes retrieval). This delay offers the ability to differentiate between those who will benefit from hCG without developing severe OHSS (the 80% false positive) and those who will not. Moreover, the observed reasonable pregnancy rate achieved, with no patient developing severe OHSS are promising, and mandate further large prospective randomized studies.

## Conclusions

COH using GnRH antagonist co-treatment and GnRHa trigger, combined with 1500 IU hCG luteal rescue, is a promising protocol, aiming to reduce (*rather than eliminating*) severe early OHSS, in one hand, and to improve reproductive outcome, in the other. However, according to the present proof of concept study, in the meantime and until further prospective randomized studies elucidating the appropriate dose and timing of HCG administration will appear, we believe that hCG luteal rescue should be offered 3 days after oocytes retrieval only to those patients with no signs of early moderate OHSS [13].

## Abbreviations

COH: Controlled ovarian hyperstimulation; ET: Embryo transfer; GnRHa: GnRH agonist; hCG: Human chorionic gonadotropin; IVF: In vitro fertilization; OHSS: Ovarian hyperstimulation syndrome.

## Competing interests

The authors declare that they have no competing interests.

## Authors’ contributions

JH- Participated in the clinical management and collected the data, performed the statistical evaluations, assisted in writing the paper and edited it in all its revisions. AK- Participated in the clinical management and edited the paper in all its revisions. RM- Participated in the clinical management and edited the paper in all its revisions. SD- Participated in the clinical management and edited the paper in all its revisions. AH- Participated in the clinical management and edited the paper in all its revisions. GY- Participated in the clinical management and edited the paper in all its revisions. RO- The principal investigator, designed the study, participated in the clinical management, performed the statistical evaluations, assisted in writing the paper and edited it in all its revisions. All authors read and approved the final manuscript.
